# Supplementary material for: Complexome profiling of the Chlamydomonas psb28 mutant reveals TEF5 as an early PSII assembly factor
Source: Plant Cell. 2025 Mar 17;37(6):koaf055. doi: 10.1093/plcell/koaf055 (PMC12164590; doi:10.1093/plcell/koaf055)
Supplement: koaf055_Supplementary_Data [file koaf055_supplementary_data.zip › psb28_interactive.zip!/plots/intensity/qualityPlots/peptides/IDH3_Cre04.g214500.t1.1.html]

### Cre04.g214500.t1.1

Trivial name: IDH3  
SeqCoverage [median]: 17.40  
Mass: 49.28 kDa  
  
 

| Sequence | MissedCleavage | Mass [Da] | wtIntensity | muIntensity | Score |
| --- | --- | --- | --- | --- | --- |
| FLQIFAETYETQYKK | 1 | 1907.97 | 648.33 | 0.00 | 86.47 |
| HAFGDQYK | 0 | 964.44 | 301.56 | 588.89 | 110.81 |
| IHVANPVVDLDGDEMTR | 0 | 1879.91 | 1090.89 | 3879.26 | 66.33 |
| IVYFDLGLPNRDK | 1 | 1548.83 | 352.84 | 0.00 | 37.43 |
| LIDDMVAQGLK | 0 | 1201.64 | 855.93 | 2331.93 | 119.68 |
| TVEAEAAHGTVTR | 0 | 1340.67 | 6024.68 | 7760.33 | 90.93 |
|  |  |  | 9274.2 | 14560.4 |  |
